# Amlodipine monotherapy vs. amlodipine–ARB combination therapy as first-line treatment for primary hypertension: a systematic review and meta-analysis

**DOI:** 10.3389/fcvm.2026.1779673

**Published:** 2026-04-22

**Authors:** Yusra Pintaningrum, Claresta Salsabila Putri Evianto, Romi Ermawan, Fitri Damayanti, R. Mohamad Javier, Kristian Kurniawan

**Affiliations:** 1 Faculty of Medicine and Health Sciences, University of Mataram, Mataram, Indonesia; 2 Division of Critical Care, Department of Cardiology & Vascular Medicine, National Cardiology Center of Harapan Kita Jakarta, Mataram, Indonesia; 3 Faculty of Medicine, Atma Jaya Catholic University of Indonesia, Jakarta, Indonesia

**Keywords:** amlodipine, combination therapy, first-line therapy, monotherapy, primary hypertension

## Abstract

**Background:**

Some clinical guidelines recommend initiating combination antihypertensive therapy as first-line treatment rather than monotherapy. Evidence indicates that a substantial proportion of patients with hypertension require more than one antihypertensive agent to achieve recommended blood pressure targets. However, it remains unclear whether the benefits of initiating combination therapy outweigh the potential risks compared with antihypertensive monotherapy.

**Objective:**

This systematic review and meta-analysis was conducted to assess the efficacy of blood pressure control and the risk of drug-related adverse events associated with amlodipine monotherapy compared against first-line combination therapy of amlodipine and an angiotensin receptor blocker (ARB) in patients with primary hypertension.

**Methods:**

A systematic literature search was conducted in PubMed, PubMed Central, and the Cochrane Library up to 15 November 2025, using the following search terms: “amlodipine” AND “angiotensin receptor blocker” AND “primary hypertension” AND “randomized controlled trial.” Only randomized controlled trials comparing amlodipine monotherapy with first-line combination therapy of amlodipine and an ARB, administered for at least 8 weeks, were included. The primary outcomes were blood pressure control and drug-related adverse events. Meta-analysis was performed using Review Manager (RevMan), version 5.4.

**Results:**

Based on six included studies, the analytical results showed that combination therapy with Calcium Channel Blocker (CCB) and an ARB was associated with 2.25 (odds ratio = 2.25: 95% CI: 1.78–2.83) times odds ratio with statistically significant overall effect (*P* < 0.00001) and 0.93 (risk ratio = 0.93: 95% CI: 0.82–1.05) times risk ratio with statistically insignificant overall effect (*P* = 0.24) compared with CCB (amlodipine 5 mg) monotherapy.

**Conclusions:**

The results of this study indicate that combination therapy with CCB and an ARB is associated with a 2.25-fold higher likelihood of achieving blood pressure control, with a significant correlation, and a lower risk of drug-related adverse events, without a significant correlation, compared with CCB monotherapy.

## 1 Introduction

In 2010, hypertension affected an estimated 1.39 billion individuals worldwide (1). Approximately 90% of cases are classified as primary hypertension, in which no identifiable secondary cause can be determined (2). Despite advances in prevention and treatment, elevated blood pressure remains the leading cause of global mortality, accounting for approximately 10.4 million deaths annually (3). Hypertension is a major risk factor for cardiovascular diseases, including stroke, myocardial infarction, renal failure, heart failure, and peripheral arterial disease. More than half of hypertensive patients also have additional cardiovascular risk factors (4).

**Figure 1:**
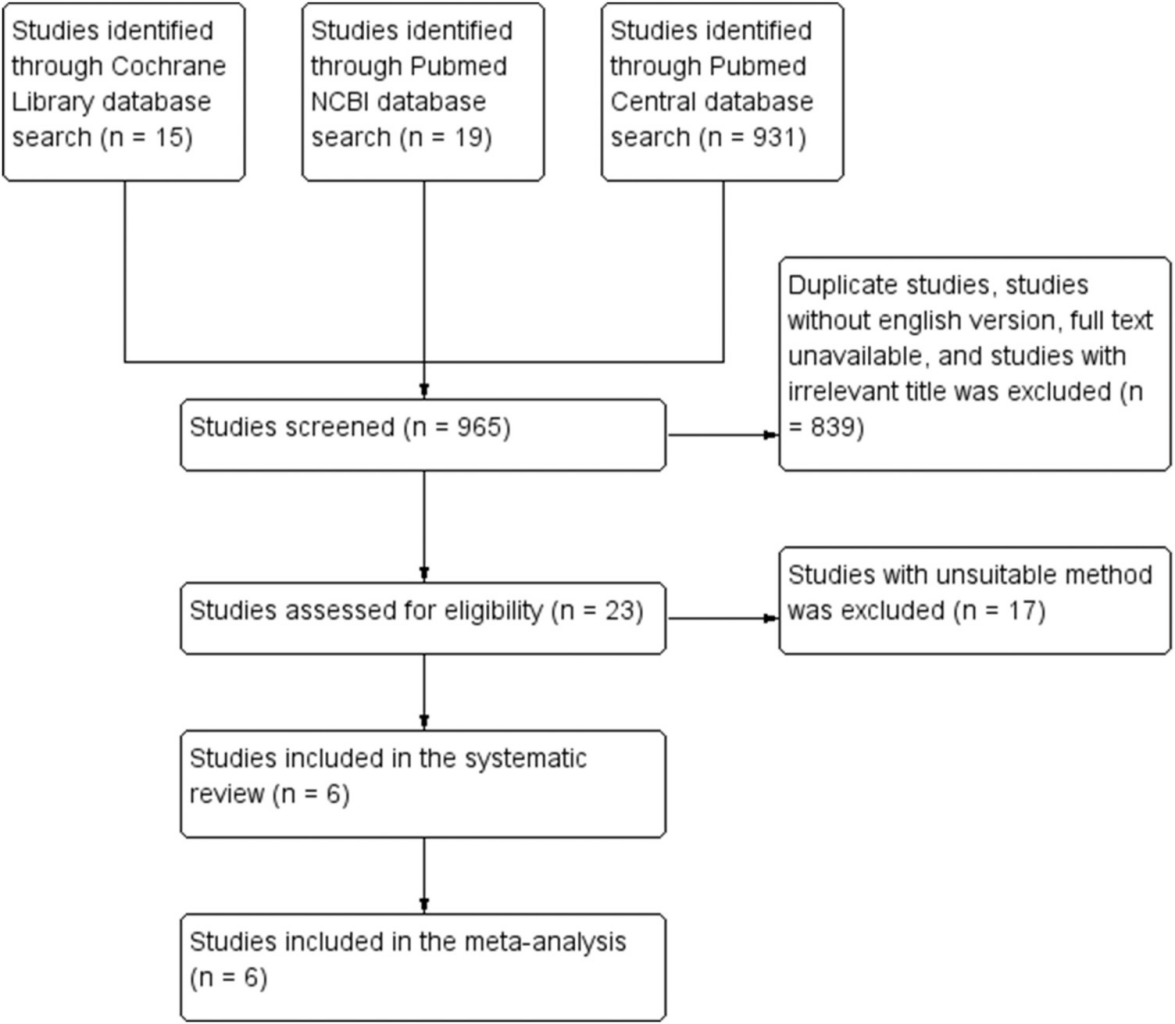
PRISMA method diagram in choosing relevant study sources.

According to major international guidelines, an office or clinic systolic blood pressure (SBP) below 130 mmHg and diastolic blood pressure (DBP) below 85 mmHg are considered normal. An SBP of 130–139 mmHg and/or a DBP of 85–89 mmHg is classified as high-normal blood pressure. Grade 1 hypertension is defined as an SBP of 140–159 mmHg and/or a DBP ≥90 mmHg, while grade 2 hypertension is defined as an SBP ≥160 mmHg and/or DBP ≥100 mmHg following repeated measurements (2). These classifications are intended to guide therapeutic strategies based on blood pressure severity.

The primary goal of antihypertensive therapy is to reduce cardiovascular morbidity and mortality without adversely affecting quality of life (5). Management strategies include both lifestyle modification and pharmacological treatment (2). Lifestyle modification plays a central role in preventing or delaying the onset of hypertension, reducing cardiovascular risk, and enhancing the effectiveness of antihypertensive medications (6). In most patients, pharmacological treatment is initiated using a stepped-care approach, in which the dose of the initial drug is increased or additional agents are introduced when blood pressure targets are not achieved (7). Recent guideline updates emphasize that the decision to initiate first-line combination antihypertensive therapy should be based on an integrated assessment of baseline blood pressure level and overall cardiovascular risk. In contrast, monotherapy is generally reserved for patients with low-risk grade 1 hypertension or very elderly individuals (>80 years) (2). Available data suggest that approximately 75% of patients with hypertension eventually require combination therapy to achieve adequate blood pressure control (8).

The rationale for initiating treatment with a combination of two antihypertensive agents is to achieve a more rapid and effective reduction in blood pressure compared with monotherapy during the early phase of treatment (7). Current guidelines preferentially recommend combinations of angiotensin-converting enzyme inhibitors or angiotensin receptor blockers (ARBs) with calcium channel blockers as first-step therapy. Alternative combinations, such as an ACEI or ARB with a thiazide-like diuretic, may be considered in specific patient populations, including very elderly individuals, post-stroke patients, those with early heart failure, or patients who are intolerant to calcium channel blockers (2).

Initiating antihypertensive treatment with low-dose combination therapy may offer several advantages, including improved blood pressure control, reduced risk of dose-related adverse effects, and better treatment adherence due to improved tolerability (9). However, potential disadvantages include unnecessary exposure to one of the agents and the risk of excessive blood pressure reduction, which may be poorly tolerated in certain individuals (10).

It remains unclear whether the benefits of initiating combination therapy for hypertension outweigh the potential harms compared with monotherapy (7). Further evaluation, especially of initial treatment with low-dose CCB and ARB combination therapy, as recommended in guidelines, compared with CCB monotherapy for primary hypertension is needed to determine the benefits and risks of this therapy.

## 2 Methods

### 2.1 Data sources and study search protocol

This systematic review and meta-analysis was conducted in accordance with PRISMA (Preferred Reporting, Items for Systematic Reviews, and Meta-Analysis) guidelines. To identify studies for this review, we searched PubMed Central, PubMed NCBI, and the Cochrane Library using the following keywords: “first line antihypertensive” AND “monotherapy” AND “combination therapy” AND “primary hypertension” AND “blood pressure control.” Only randomized controlled trials with a minimum of 50 participants per treatment arm and a follow-up duration of at least 8 weeks were included. Controlled blood pressure was defined as a systolic blood pressure <140 mmHg and/or a diastolic blood pressure <90 mmHg at the end of follow-up.

### 2.2 Study selection and quality assessment

We selected quantitative studies published within the late 20 years that compared CCB monotherapy with CCB + ARB combination therapy for at least 8 weeks as first-line treatment for primary hypertension patients to assess the percentage of patients achieving blood pressure control and the incidence of therapy-related adverse events for both treatment strategies. The CCB included in this study was amlodipine 5 mg, used either as monotherapy or in combination with a dosage-equivalent ARB, including losartan 50 mg, temilsartan 40 mg, olmesartan 20 mg, valsartan 80 mg, and candesartan 16 mg. The primary efficacy endpoint was the proportion of patients achieving controlled blood pressure, defined as systolic blood pressure <140 mmHg and/or diastolic blood pressure <90 mmHg at the end of follow-up, which was the most consistently reported threshold across the included trials.

The literature search was conducted on 15 November 2025 using PubMed, PubMed Central, and the Cochrane Central Register of Controlled Trials. The following search strategy was applied in PubMed: “amlodipine” AND “angiotensin receptor blocker” AND “hypertension” AND “randomized controlled trial.” Equivalent search terms were adapted for other databases. Clinical trial registries, including ClinicalTrials.gov and the WHO International Clinical Trials Registry Platform, were also searched to identify ongoing or unpublished trials. However, no additional eligible studies were identified. This initial search yielded 965 hits, of which 839 were excluded due to irrelevant titles, duplication, lack of an English version, and unavailable full text. The remaining 23 studies were further assessed to determine whether they met specific inclusion criteria; of these, 17 studies were excluded, leaving a final sample of six relevant studies that serve a representative purpose for this analytical review. For quality assessment of the study, we manually assessed the (1) risk of bias, (2) blinding, (3) loss of data, (4) selective reporting of the study result, (5) study design, (6) study personnel, (7) result analysis methods, and (8) other potential sources of bias. Drug-related adverse events assessed in this review included peripheral edema, dizziness, headache, hypotension, cough, hyperkalemia, and treatment discontinuation due to adverse effects, as reported in the original trials.

### 2.3 Data synthesis and statistical analysis

Data from the included studies were pooled to estimate the odds ratio for achieving controlled blood pressure and the risk ratio for therapy-related adverse events, comparing calcium channel blocker monotherapy with calcium channel blocker plus angiotensin receptor blocker combination therapy as first-line treatment in patients with primary hypertension. For the efficacy analysis of blood pressure control, only treatment arms using amlodipine 5 mg monotherapy and amlodipine 5 mg combined with an ARB were included to ensure dose comparability across trials and to minimize dose-related confounding. In contrast, the safety analysis of drug-related adverse events included all available treatment arms, regardless of dose variation, as several trials reported adverse events across multiple dosing regimens rather than exclusively within the fixed-dose subgroup.

Statistical analysis was performed using the Mantel–Haenszel method under a fixed-effects model (11), with patients considered the unit of analysis. Effect estimates were expressed as odds ratios (ORs) and risk ratios (RRs) with corresponding 95% confidence intervals (CIs) (11). Between-study heterogeneity was assessed using the Cochran *Q* test and the *I*² statistic, with heterogeneity considered statistically significant when *P* < 0.05 or *I*^2^ > 70% (12). *I*^2^ values of 25%, 50%, and 75% were interpreted as low, moderate, and high heterogeneity, respectively. A two-sided alpha level of 0.05 was considered statistically significant.

The risk of bias in the included randomized controlled trials was independently assessed by two reviewers using the Cochrane Risk of Bias 2.0 tool. The following domains were evaluated: bias arising from the randomization process, deviations from intended interventions, missing outcome data, measurement of outcomes, and selection of the reported result. Discrepancies were resolved through discussion. To evaluate whether any individual study influenced the pooled results, a leave-one-out sensitivity analysis was performed by sequentially excluding each study and recalculating the pooled effect estimates.

When heterogeneity was not statistically significant, a fixed-effects model was applied; otherwise, a random-effects model was used, followed by subgroup analysis (12). Sensitivity analysis was performed by sequentially excluding individual studies. Publication bias was assessed visually using funnel plots. All analyses were conducted using Review Manager software (RevMan), version 5.4.

## 3 Results

### 3.1 Demographic characteristics of the included studies

Funding sources were reported in the majority of included trials, with most studies supported by academic or governmental institutions and no consistent evidence of industry sponsorship bias. Overall, the included trials demonstrated a low to moderate risk of bias across most domains. Random sequence generation and outcome measurement were generally well described, although some concerns were noted regarding allocation concealment and blinding in a minority of studies. No study was judged to be at high risk of bias across multiple domains. We identified 965 studies through the keyword search, and after screening and eligibility assessment, six studies were considered potentially relevant for this analytical review. Seventeen full-text articles were excluded for the following reasons: non-randomized design (*n* = 7), insufficient outcome data (*n* = 5), and duplicate or overlapping study populations (*n* = 5). All of the potentially relevant studies were randomized controlled trials, with a minimum duration of 8 weeks, and were placebo-controlled; of these, five were double-blinded and one was single-blinded. The six included studies comprised a total of 6,401 participants from 24 countries all across the world, including Belgium, Canada, France, Germany, Mexico, the United States, Egypt, Norway, Korea, Peru, Malaysia, Portugal, Spain, Taiwan, South Africa, Argentina, Brazil, Finland, Italy, the Netherlands, the United Kingdom, Poland, Russia, and Ukraine. The mean age of the participants ranged from approximately 53.1 to 56.9 years oldwith a mean baseline SBP ranging from 153 to 169 mmHg and a mean baseline DBP ranging from 93 to 102 mmHg. The demographic characteristics and outcome of the included studies are summarized in Table 1. The angiotensin receptor blockers used across the included trials included losartan, valsartan, telmisartan, and olmesartan, administered at standard therapeutic doses as defined by each study protocol. Sensitivity analysis demonstrated that the removal of any single study did not materially change the overall pooled estimate, indicating that the results were not driven by any individual trial.

**Table 1:** Demographic and outcome characteristics of the included studies.

Studies	Study location	Study method	Study duration (weeks)	Blinding	Baseline mean BP (mmHg)	Mean age (years)	Participants (*n*)	Outcome				NNT	NNH
								Controlled BP (event/total)		Therapy-related adverse event (event/total)			
								AML 5 mg	AML 5 mg + ARB Equivalent Dose	AML	AML + ARB		
Phillip et al. (13)	Belgium, Canada, France, Germany, Mexico, United States, Egypt, Norway, Korea, Peru, Malaysia, Portugal, Spain, Taiwan	RCT	8	Double-blind	156/99	56.9	1,911	92/128	109/128	115/460	288/1,437	-	-
Chrysant et al. (14)	United States	RCT	8	Double-blind	164/102	54.0	1,940	34/121	68/160	86/324	268/970	-	-
Littlejohn et al. (15)	United States, South Africa, Argentina, Brazil	RCT	8	Double-blind	153/101	53.1	1,461	58/137	83/141	39/309	102/765	-	-
Volpe et al. (16)	Belgium, Finland, Italy, Germany, Netherlands, Unites Kingdom, Poland, Russia, Ukraine	RCT	8	Double-blind	164/102	55.8	755	40/68	89/118	14/188	38/567	-	-
Kim et al. (17)	Korea	RCT	8	Double-blind	169/102	55.3	149	69/75	71/73	13/75	8/73	-	-
Punzi et al. (18)	United States	RCT	16	Single-blind	158/93	56.9	185	44/185	85/179	5/185	9/180	-	-
							6,401	337/714 (47.20%)	505/799 (63.20%)	272/1,541 (17.65%)	713/3,992 (17.86%)	6	476

AML, amlodipine; ARB, angiotensin receptor blocker; AE, adverse event; BP, blood pressure; RCT, randomized controlled trial; NNT, number needed to treat; NNH, number needed to harm.

### 3.2 Outcome characteristics of the included studies

A total of 6,401 participants were included across the six studies evaluating first-line therapy for primary hypertension with CCB monotherapy versus CCB and ARB combination therapy. To assess therapy-related adverse events, 5,533 participants were included; of these, 1,541 (27.8%) received CCB monotherapy with varying doses, while 3,992 (72.2%) received CCB and ARB combination therapy with varying doses. Among patients receiving varying doses of CCB monotherapy, 272 (17.65%) of 1,541 patients reported therapy-related adverse events, compared with 713 (17.86%) of 3,992 patients receiving varying doses of CCB and ARB combination therapyat least 8 weeks of therapy. For the analysis of blood pressure control, only participants receiving amlodipine 5 mg monotherapy or amlodipine 5 mg in combination with an ARB were included. In contrast, the safety analysis of drug-related adverse events included participants receiving variable doses of amlodipine and ARBs, reflecting how safety outcomes were reported in the original trials.

Among these 1,513 participants, 714 (47.1%) received a specific dose of CCB monotherapy (amlodipine 5 mg), while 799 (52.9%) received a specific dose of CCB monotherapy (amlodipine 5 mg) combined with an ARB (equivalent dose of losartan 50 mg/olmesartan 20 mg/telmisartan 40 mg/valsartan 80 mg/candesartan 18 mg) for the assessment of blood pressure control. Among patients receiving amlodipine 5 mg, 337 (47.20%) of 714 patients achieved controlled blood pressure, compared with 505 (63.20%) of 799 patients in the combination therapy group after at least 8 weeks of treatment. The outcome characteristics of the included studies are summarized in Table 1.

### 3.3 Efficacy of CCB monotherapy compared with CCB and ARB combination therapy toward blood pressure control in primary hypertension patients

**Figure 2:**
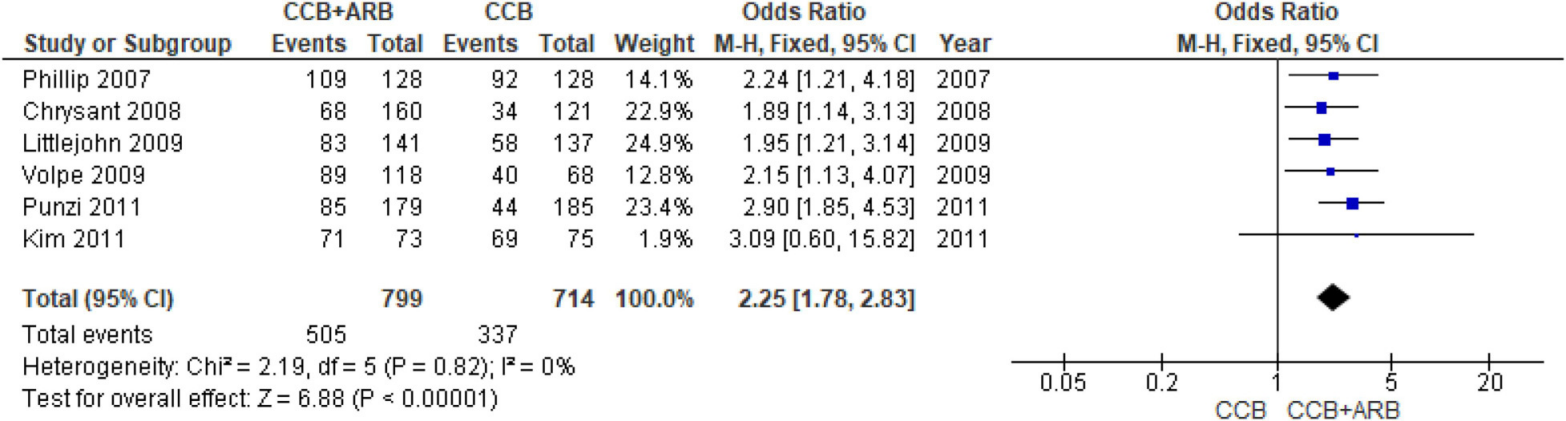
Forest plot of the odds ratio for blood pressure control comparing CCB monotherapy with CCB and ARB combination therapy as first-line treatment in primary hypertension.

The meta-analysis forest plot presented in Figure 2 showed heterogeneity (*I*^2^ = 0%; *χ*^2^ = 2.19; *P* = 0.82). As the six included studies were statistically homogeneous, based on heterogeneity analysis results, we conducted a summary effect measure to evaluate the odds ratio of blood pressure control outcome from CCB monotherapy (amlodipine 5 mg) versus CCB (amlodipine 5 mg) and ARB (equivalent dose of losartan 50 mg/olmesartan 20 mg/telmisartan 40 mg/valsartan 80 mg/candesartan 18 mg) combination therapy. The analytical results indicate that CCB and ARB combination therapy was associated with a 2.25 (OR = 2.25: 95% CI: 1.78–2.83) times odds ratio, with a statistically significant overall effect (*P* < 0.00001) compared with CCB monotherapy. This result indicates a 2.25-fold higher likelihood of achieving controlled blood pressure with CCB and ARB combination therapy compared with CCB monotherapy.

**Figure 3:**
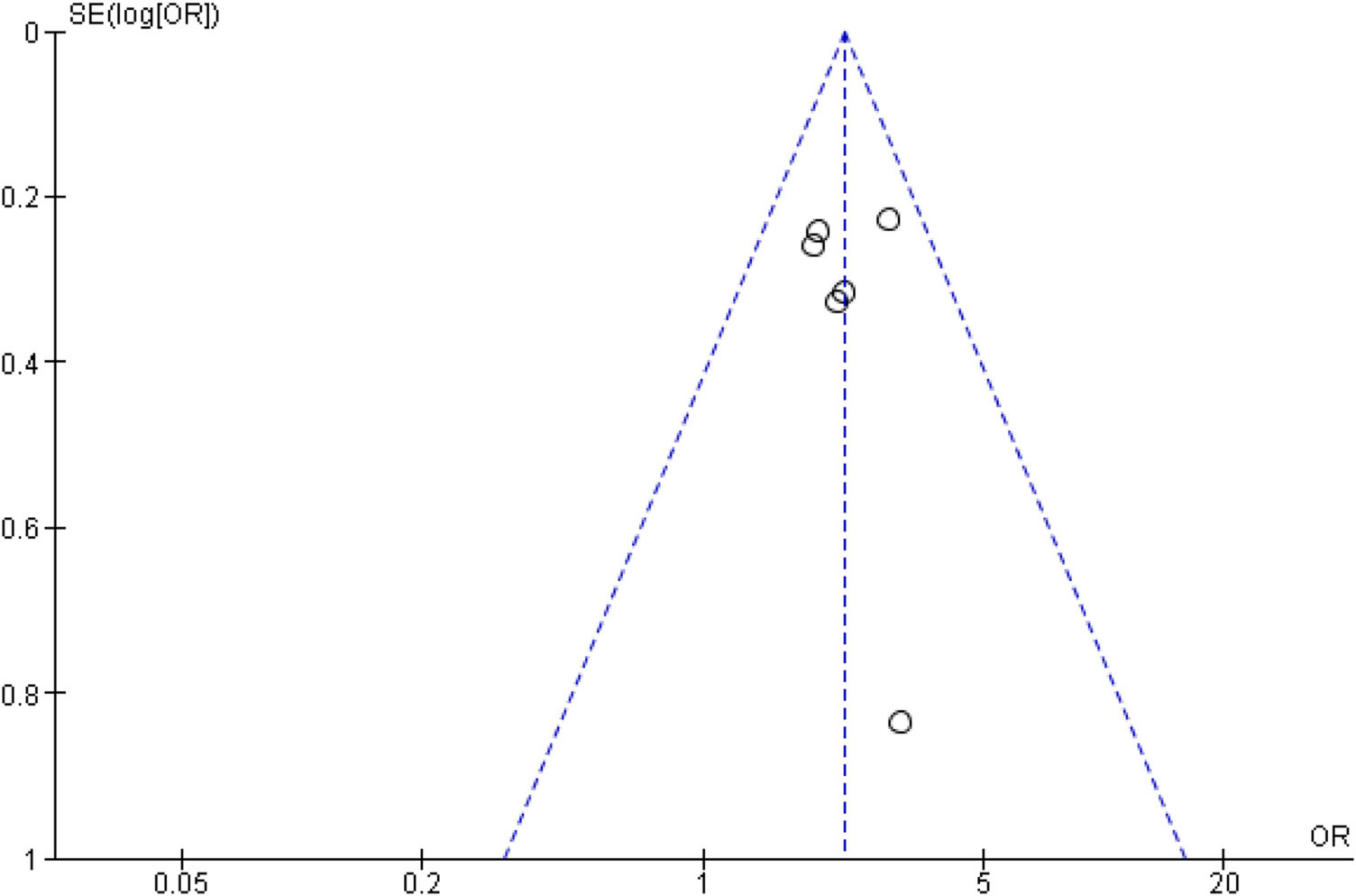
Funnel plot of the odds ratio for blood pressure control comparing CCB monotherapy with CCB and ARB combination therapy as first-line treatment in primary hypertension.

To assess the risk of publication bias, a funnel plot assessment was performed. Visual inspection of the funnel plot (Figure 3) showed asymmetry among the six included studies. However, given that only six studies were included, the power of the funnel plot to detect publication bias is limited. Therefore, the risk of publication bias should be considered possible but inconclusive, rather than definitively high.

### 3.4 CCB monotherapy compared with CCB with ARB combination therapy for drug-related adverse event risk

**Figure 4:**
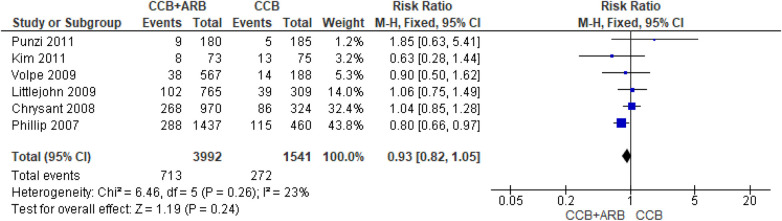
Forest plot of the risk of drug-related adverse events comparing CCB monotherapy with CCB and ARB combination therapy as first-line treatment in primary hypertension.

The meta-analysis forest plot presented in Figure 4 showed heterogeneity (*I*^2^ = 23%; *χ*^2^ = 6.46; *P* = 0.26). As the six included studies were statistically homogenous, based on heterogeneity analysis results, we conducted a summary effect measure to evaluate the risk of drug-related adverse events for CCB monotherapy (variable doses of amlodipine) compared with CCB (variable doses of amlodipine) and ARB (variation dose of losartan/olmesartan/telmisartan/valsartan/candesartan) combination therapy. The analytical results showed a risk ratio of 0.93 (RR = 0.93: 95% CI: 0.82–1.05), with statistically insignificant overall effect (*P* = 0.24), for CCB and ARB combination therapy compared with CCB monotherapy. This result indicates no statistically significant difference in the risk of drug-related adverse events between amlodipine monotherapy and amlodipine–ARB combination therapy.

**Figure 5:**
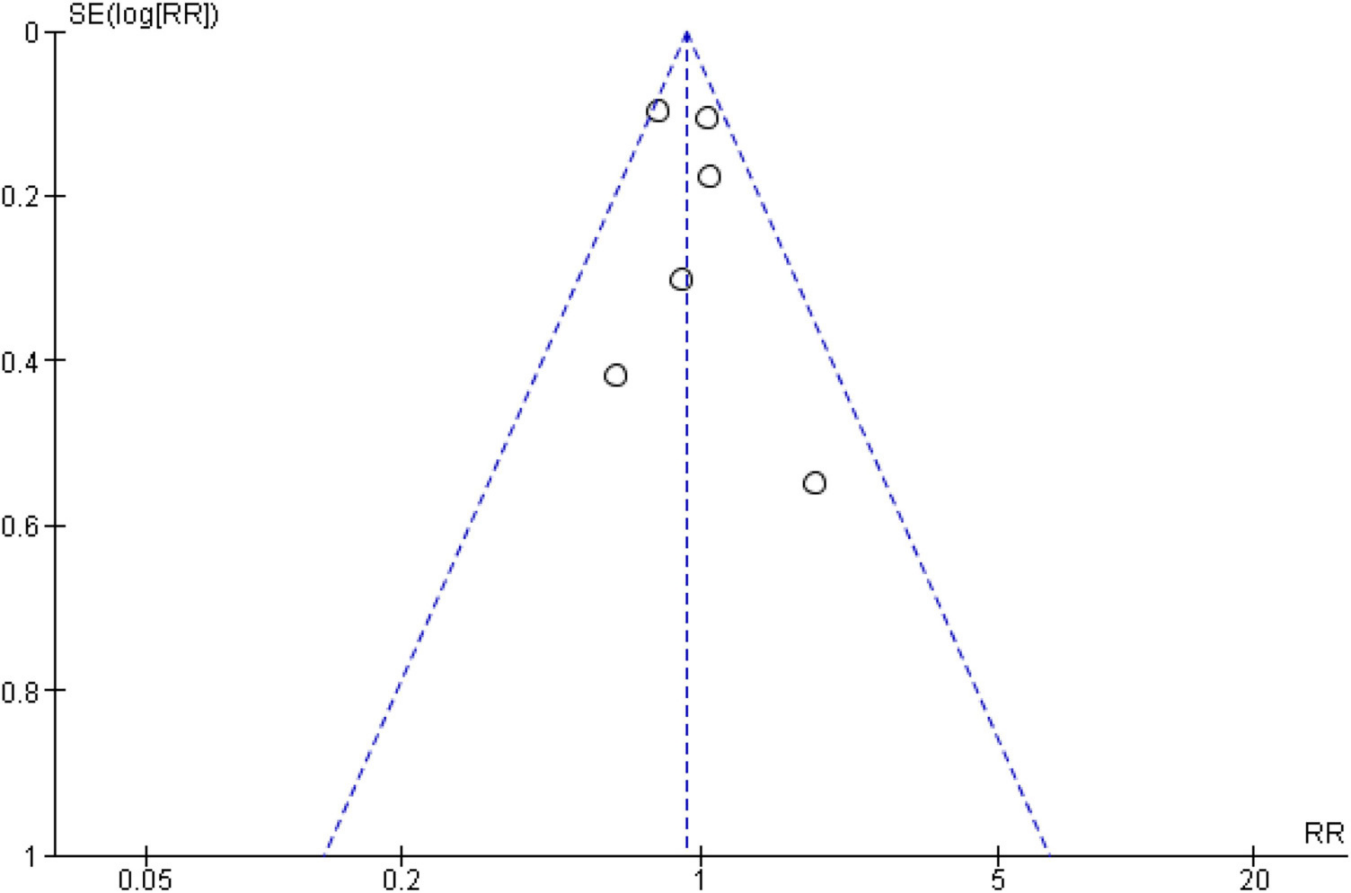
Funnel plot of the odds ratio for primary hypertension blood pressure control comparing CCB monotherapy with CCB and ARB combination therapy.

To assess the risk of publication bias, a funnel plot assessment was performed. Visual inspection of the funnel plot (Figure 5) demonstrated symmetry among the six included studies. However, because only six studies were included, the statistical power of the funnel plot to detect publication bias is limited; therefore, publication bias cannot be reliably assessed.

## 4 Discussion

Although current hypertension guidelines recommend combination antihypertensive therapy for many patients, direct meta-analytic evidence specifically comparing amlodipine monotherapy with amlodipine–ARB combination therapy as first-line treatment remains limited.

This study provides a focused synthesis of randomized controlled trials evaluating this specific therapeutic strategy and offers clinically relevant evidence on the incremental benefit of adding an ARB to amlodipine in the early management of primary hypertension.

Our findings are consistent with the Cochrane review by Garjón et al. (7), which reported that initial combination therapy is more effective in achieving blood pressure control than monotherapy, although evidence on long-term cardiovascular outcomes remains limited (7). Similar findings were also reported by Law et al. (BMJ 2009), who demonstrated greater blood pressure reduction with combination regimens compared with single-drug therapy (5). However, unlike some earlier studies, the present analysis focused exclusively on randomized controlled trials and directly compared amlodipine monotherapy with amlodipine combined with an ARB as first-line treatment for primary hypertension. Individuals with confirmed hypertension (grade 1 or grade 2) should receive appropriate pharmacological treatment (2); however, there is variation among guidelines regarding whether blood pressure-lowering drugs should be initiated in grade 1 hypertension with low-to-moderate CV risk, in older patients (>60 years) with grade 1 hypertension, or in patients with high-normal blood pressure levels (9).

The 2020 International Society of Hypertension (ISH) guidelines suggest that the determination of the need for first-line combination antihypertensive drug therapy should be based on a combined assessment of the blood pressure level and the risk for cardiovascular disease. In contrast, first-line antihypertensive drug monotherapy is only considered for patients with low-risk grade 1 hypertension or for very elderly patients (≥80 years) (2). Other major guidelines support the same recommendation. The 2018 guidelines from the American College of Cardiology (ACC) and the American Heart Association (AHA), along with several other organizations, recommend the initiation of antihypertensive therapy with two first-line agents from different classes in adults with stage 2 hypertension (19). Patients with confirmed hypertension (grade 1 or grade 2) are generally recommended to receive pharmacological treatment (2). However, variations exist among clinical guidelines regarding the initiation of antihypertensive therapy in patients with grade 1 hypertension and low-to-moderate cardiovascular risk, in older individuals, or in those with high-normal blood pressure levels (9, 19, 20).

We analyzed the proportion of patients achieving blood pressure control and the risk of drug-related adverse events for CCB monotherapy compared with CCB and ARB combination therapy. Statistical analysis showed that CCB (amlodipine 5 mg) and ARB (equivalent dose of losartan 50 mg/olmesartan 20 mg/telmisartan 40 mg/valsartan 80 mg/candesartan 18 mg) combination therapy showed statistically significant correlation with a 2.25-fold higher likelihood of achieving controlled blood pressure compared with CCB monotherapy. Statistical analysis comparing CCB monotherapy with CCB and ARB combination therapy showed no statistically significant difference in the incidence of drug-related adverse events between the two treatment strategies. Although calcium channel blocker monotherapy has been associated with a higher incidence of peripheral edema in prior trials, this meta-analysis did not demonstrate a statistically significant increase in drug-related adverse events. This discrepancy may be explained by the use of combination therapy, as ARBs are known to mitigate CCB-induced edema through postcapillary venodilation. In addition, adverse event reporting varied across studies and may have been underpowered to detect differences in less frequent safety outcomes. In the study by Philipp et al. (13), peripheral edema occurred more frequently in the amlodipine monotherapy group than in the amlodipine–valsartan combination group (13). Similarly, the COACH trial (14) demonstrated a lower incidence of peripheral edema with olmesartan–amlodipine combination therapy compared with amlodipine alone (14).

Based on pooled absolute risk differences, the estimated number needed to treat (NNT) was 6, indicating that approximately six patients would need to receive amlodipine–ARB combination therapy for one additional patient to achieve controlled blood pressure compared with amlodipine monotherapy.

The estimated number needed to harm (NNH) was 476, indicating that adverse events occurred at very similar rates between the treatment groups and suggesting comparable safety profiles.

The findings of this study are in line with recommendations from the ISH, ACC/AHA, and ESH/ESC, which suggest that the need for first-line combination antihypertensive drug therapy should be based on a combined assessment of the blood pressure level and the risk for cardiovascular disease. In contrast, first-line antihypertensive drug monotherapy is considered only for patients with low-risk grade 1 hypertension or for very elderly patients (≥80 years) (2, 9, 19).

Despite the advantages of combination therapy, antihypertensive monotherapy may still have a role in selected patient populations, such as individuals with mild hypertension, older adults with polypharmacy concerns, or those in resource-limited settings. Treatment decisions should therefore be individualized based on patient characteristics and the healthcare context.

These findings suggest that initial combination therapy with amlodipine and an ARB may offer improved blood pressure control compared with monotherapy in selected patients. However, further large-scale trials with longer follow-up are warranted to confirm long-term cardiovascular outcomes and safety. Publication bias represents an important limitation of this meta-analysis. Visual inspection of the funnel plot for the primary outcome suggested asymmetry, raising the possibility that smaller studies with neutral or negative findings may be underrepresented. This could have resulted in an overestimation of the pooled treatment effect. Accordingly, the magnitude of the observed benefit should be interpreted with caution. While improved blood pressure control is a well-established surrogate for reduced cardiovascular risk, the included trials were not powered to assess major adverse cardiovascular events or mortality. Future large-scale studies with longer follow-up are required to determine whether the observed blood pressure benefits translate into improved hard cardiovascular outcomes.

## 5 Conclusions

This meta-analysis demonstrated that amlodipine combined with an angiotensin receptor blocker was associated with a significantly higher likelihood of achieving blood pressure control compared with amlodipine monotherapy. However, no statistically significant difference in the risk of drug-related adverse events was observed between the two treatment strategies. These findings suggest that initiating treatment with an amlodipine–ARB combination may improve blood pressure control without increasing the risk of adverse events.

## Data Availability

The original contributions presented in the study are included in the article/Supplementary Material, further inquiries can be directed to the corresponding author.
